# Perillyle alcohol and Quercetin ameliorate monocrotaline-induced pulmonary artery hypertension in rats through PARP1-mediated miR-204 down-regulation and its downstream pathway

**DOI:** 10.1186/s12906-020-03015-1

**Published:** 2020-07-13

**Authors:** Soodeh Rajabi, Hamid Najafipour, Saeideh Jafarinejad Farsangi, Siyavash Joukar, Ahmad Beik, Maryam Iranpour, Zeinab Kordestani

**Affiliations:** 1 PhD candidate, Department of Physiology and Pharmacology, and Physiology Research Center, Institute of Basic and Clinical Physiology Sciences, Kerman University of Medical Sciences, Kerman, Iran; 2 Cardiovascular Research Center, Institute of Basic and Clinical Physiology Sciences, Kerman University of Medical Science, Kerman, Iran; 3 Department of Physiology and Pharmacology, Afzalipour Medical Faculty, Bulvd. 22 Bahman, Kerman, Iran; 4 Molecular Biology, Physiology Research Center, Institute of Basic and Clinical Physiology Sciences, Kerman University of Medical Science, Kerman, Iran; 5 Department of Pathology and Pathology and Stem Cell Research Center, Kerman University of Medical Sciences, Kerman, Iran; 6 Researcher, Endocrinology and Metabolism Research Center, Kerman University of Medical Sciences, Kerman, Iran

**Keywords:** Pulmonary artery hypertension, miR-204, Perillyle alcohol, Quercetin, PARP1, HIF1a, NFATc2

## Abstract

**Background:**

Pulmonary artery hypertension (PAH) is a vascular disease in the lung characterized by elevated pulmonary arterial pressure (PAP). Many miRNAs play a role in the pathophysiology of PAH. Perillyle alcohol (PA) and Quercetin (QS) are plant derivatives with antioxidant and anti-proliferative properties. We investigated the effect of PA and QS on PAP, expression of PARP1, miR-204, and their targets, HIF1α and NFATc2, in experimental PAH.

**Methods:**

Thirty rats were divided into control, MCT, MCT + Veh, MCT + PA and MCT + QS groups. MCT (60 mg/kg) was injected subcutaneously to induce PAH. PA (50 mg/kg daily) and QS (30 mg/kg daily) were administered for 3 weeks after inducing PAH. PAP, lung pathology, expression of miRNA and mRNA, and target proteins were evaluated through right ventricle cannulation, H&E staining, real-time qPCR, and western blotting, respectively.

**Results:**

Inflammation and lung arteriole thickness in the MCT group increased compared to control group. PA and QS ameliorated inflammation and reduced arteriole thickness significantly. miR-204 expression decreased in PAH rats (*p* < 0.001). PA (p < 0.001) and QS (*p* < 0.01) significantly increased miR-204 expression. Expression of PARP1, HIF1α, NFATc2, and α-SMA mRNA increased significantly in MCT + veh rats (all *p* < 0.001), and these were reduced after treatment with PA and QS (both *p* < 0.01). PA and QS also decreased the expression of PARP1, HIF1α, and NFATc2 proteins that had increased in MCT + Veh group.

**Conclusion:**

PA and QS improved PAH possibly by affecting the expression of PARP1 and miR-204 and their downstream targets, HIF1a and NFATc2. PA and QS may be therapeutic goals in the treatment of PAH.

## Background

Pulmonary artery hypertension (PAH) is a chronic disease of the pulmonary vasculature characterized by elevated pulmonary arterial pressure (PAP) due to remodeling and vasoconstriction in small arterioles, leading to right ventricular failure and ultimately death [1, 2]. The pathophysiological processes that occur in PAH include inflammation, fibrosis, and vascular remodeling due to increased proliferation and reduced apoptosis, leading to narrowing of vessels lumen and increase in vascular resistance [2].

Despite many efforts to understand and treat PAH, there is still no definite cure for it, and most vascular dilator therapies have not led to full recovery, with patients dying in less than a decade depending on the severity of the disease [3]. Therefore efforts are ongoing to understand the molecular pathways and mechanisms involved in the pathophysiology of PAH and finding more effective therapies.

Micro-RNAs (miRNAs) are small non-coding RNAs that are dysregulated in many diseases [4, 5]. These small single-stranded RNAs are about 22 nucleotides in length and destroy/suppress translation of their target mRNAs by binding to them, thereby affecting the expression of the target genes [5]. Many miRNAs play a role in the pathophysiology of PAH [6, 7]. MiR-204 is one of the important miRNAs involved in PAH and cancers [8, 9]. In a study on colorectal cancer, miR-204 suppressed tumor cell proliferation and invasion and increased the susceptibility of the cells to chemical treatments [10].

Chronic inflammation and proliferation of pulmonary arterial smooth muscle cells (PASMCs) and reduced apoptosis are events that accelerate the progression of disease in PAH [11]. In a number of studies in human and animal models of PAH, it has been found that the level of miR204 decreases in PASMCs [11, 12]**.** It has also been shown that the activation of Poly ADP-ribose polymerase-1 (PARP1) due to inflammation results in pulmonary hypertension associated with decrease in miR-204 levels. In fact, severe inflammation caused by the disease results in DNA damage and activates PARP1 [13]. PARP1 activation repairs damaged DNA and leads to increased proliferation [9]. Meloch et al. showed that PARP1 inhibitor (ABT-888) could reverse PAH-induced abnormalities in vivo [9]. On the other hand, miR-204 down-regulation induces hypoxia-inducible factor 1-alpha (HIF1α) and nuclear factor of activated T-cells cytoplasmic 2 (NFATc2) activation, resulting in the anti-apoptotic and, therefore, proliferative phenotype of PAH [14].

Nowadays, the use of plant derivatives has attracted researchers due to the relatively fewer side effects and better efficacy in disease treatment resulting from their simultaneous impact on several aspects of the disease. Derivatives such as perillyle alcohol (PA), a monotrepene mostly found in mint and cherries, and quercetin (QS), a flavonoid mostly found in grapes and berries, are noteworthy for their anti-oxidant and anti-proliferative properties. Currently QS is marketed as a dietary supplement in several countries [15]. The protective effect of QS against cancer is due to its effect on miRNAs and their targets [16, 17]. Sonoki et al. showed that in lung adenocarcinoma cell lines, QS decreases claudin-2 expression through up-regulation of miRNA-16, which leads to reduction in proliferation and migration in lung cancer [18].

In two studies on hepato-carcinogenesis in rats, treatment with PA prevented oxidative stress and reduced cancer incidence [19, 20]. Although the role of PA has been investigated in different cancer cell lines and has shown anti-tumor, anti-oxidant, and anti-proliferative effects, its effect on PAH and its molecular pathways has not been investigated. Also, the mechanism of the ameliorative effect of QS in PAH through miR-204 and PARP1 has not been studied so far.

As some features of PAH such as proliferation and inhibition of apoptosis have a cancer-like pattern, and PA and QS have shown to have anti-cancer effects, we examined the effect of these plant derivatives on the treatment of PAH in a rat model. The use of an animal model permits us to harvest the lung and heart tissues for pathological investigation purposes and assess the underlying mechanisms including expression of PARP1 and miR-204 and their downstream targets, HIF1a and NFATc2, in the diseased lung.

## Methods

### Animals

**Fig. 1:**
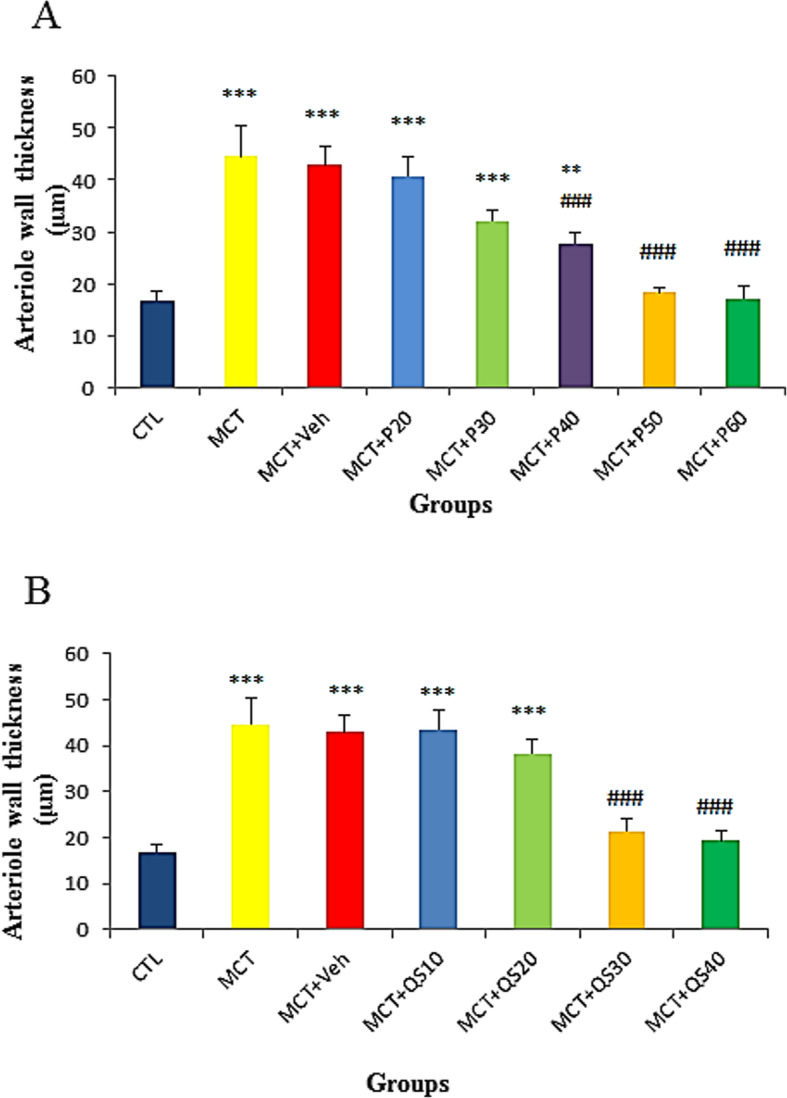
Dose-response curve for determining the optimum dose of PA (**a**) and QS (**b**) to reduce pulmonary arteriole wall thickness (*n* = 5 in each group). For PA, dose of 50 mg/kg and for QS, dose of 30 mg/kg were selected for the main experiments. Values are mean ± SD. CTL, control; MCT, monocrotaline; Veh, vehicle; PA, perillyle alcohol; QS, quercetin

Male Wistar rats weighting 220–280 g (8–10 weeks of age) provided by the animal house of Kerman Physiology Research Center were kept under a 12–12 h lights on-off cycle and 23 ± 2 °C temperature with free access to water and food. The experiment protocol was approved by the Ethics Committee of Kerman University of Medical Sciences, Iran (Permission No. IR.KMU.REC.1395.244). Sixty rats were randomly divided into 12 groups (*n* = 5) for pilot dose-response studies to determine the optimum dose of PA and QS (see Fig. 1 in result section). Thirty other rats were randomly divided into five main groups (*n* = 6): Control (CTL), Monocrotaline (MCT), MCT + vehicle (Veh), MCT + Perillyle alcohol (PA), and MCT + quercetin (QS). If an animal was lost during the study, another animal was entered in the study from the beginning of the protocol to fulfill the determined sample size in each group.

### Induction of PAH

PAH was induced by a subcutaneous single dose (60 mg/kg) injection of MCT [21] (Sigma-Aldrich Co., St. Louis, MO, USA) in a volume of 0.2 ml at day 0. The control group received 0.2 ml normal saline in day 0, subcutaneously. After 3 weeks, when rats developed PAH, they received intra-peritoneal daily injections of saline, 5% ethanol, 50 mg/kg PA or 30 mg/kg QS for 3 weeks (from day 22 to day 42) [21]. These doses of PA and QS were selected based on the pilot dose-response study (see Fig. 1 in result section).

### Right ventricular pressure and hypertrophy measurements

At the end of the experiments (day 43), the rats were anaesthetized by intra-peritoneal injection of sodium thiopental (50 mg/kg). The level of anesthesia was assured by the absence of withdrawal response to a pinch stimulus applied to the paw of the animal, and if necessary, a complementary dose of anesthesia (10 mg/kg sodium thiopental, ip) was injected. A polyethylene catheter (PE-50) was inserted through the right jugular vein into the right ventricle to measure the right ventricular systolic pressure (RVSP) with the Powerlab Physiograph system (AD Instruments, Sydney, Australia). After recording RVSP, rats were euthanized under deep anesthesia (200 mg/kg sodium thiopental, ip), and the chest was opened to remove the heart and lungs for pathological and/or molecular studies. The heart right ventricle (RV) and left ventricle + septum (LV + S) were weighed. RVSP and RV/LV + S weight ratio were reported as indices of pulmonary artery systolic pressure and RV hypertrophy, respectively.

### Tissue preparation and histopathology assessment of the lung

Immediately after removal of the lungs, a portion was frozen in liquid nitrogen and stored at − 80 °C. These tissues were later homogenized and used to assess the expression of mRNA and protein levels of PARP1, miR-204, HIF1α, and NFATc2 in the supernatant (see below). Another portion was fixed with 10% buffered formalin (pH 7.4), embedded in paraffin, and cut into 5 μm-thick sections. The sections were stained with hematoxylin and eosin (H & E) and examined microscopically by a pathologist who was blind to the animal groups. For investigation of inflammation, 6 fields in each sample were selected. The scoring system for quantification of inflammation was from 0 to 3 based on absence (no), mild (< 20% of the lung), moderate (20–50% of the lung) or severe (> 50% of the lung) inflammation [19, 20].

Arteriole wall thickness was determined by computer light microscope (Olympus, Japan) by subtracting the internal diameter from the external diameter of the arteriole. For each specimen, an average of the diameter of ten randomly selected arterioles in the range of 50–120 μm was calculated.

### Assessment of inflammatory factors in lung tissue

For assessing lung inflammation, the level of IL-1β and IL-8, important cytokines in PAH, were measured [22]. The amounts of IL-1β (Cat number: DY501, R&D Systems) and IL-8 (Cat number: MBS025179, Mybiosource) were measured in the lung homogenates with an enzyme-linked immunosorbent assay. In brief, known concentrations of recombinant rat IL-1β or IL-8 and the experimental samples were added and incubated in polystyrene microtiter plates coated with an antibody against the appointed cytokine, followed by incubation with an enzyme-linked polyclonal antibody directed to the cytokine. Next, a substrate solution for the enzyme was added, and the color development was stopped by adding 2 N H2SO4. The absorbance was measured with a microtiter plate spectrophotometer. The amount of IL-1β, or IL-8 present in each sample was determined from a standard curve generated in each assay and expressed as picograms per milliliter. The sensitivities of the enzyme-linked immunosorbent assays for IL-1β and IL-8 were 5 pg/ml and 2.0 pg/ml, respectively.

### miRNA isolation and quantitative real-time PCR

The lung tissues were homogenized with an ultrasonic homogenizer (UP 200H, Germany), and total RNA (enriched for small RNA) was extracted according to the manufacturer’s instructions using total RNA Mini-Preps Kit (Bio Basic, Canada). RNA concentration and purity were quantified using NanoDrop ND-2100 (Thermo Fisher Scientific, USA). Complementary DNA (cDNA) syntheses of miR-204 and target genes were performed using the PrimeScript 1st cDNA Synthesis Kit (Takara Bio, Japan). For miR-204 cDNA synthesis, RT primer was added to the reaction mixture.

Using a miRNA-specific stem-loop primer, expression of miRNA-204 was quantified using Ampliqon master mix (StepOnePlus instrument, Applied Biosystems, USA). Small nucleolar RNA U6 (RNU6) and 18 s rRNA were used as internal controls for miR-204 and target genes (PARP1, HIF1α, NFATc2, and α-SMA), respectively. The primer sequences of miRNA-204 and target genes are reported in Table 1.

**Table 1:** Primer sequences of miRNA-204 and target genes

miRNA-204	RT	5′-GTTGGCTCTGGTGCAGGGTCCGAGGTATTCGCACCAGAG CCAACAGGCAT-3’	[23]
	Forward	5′-GCGGCGGTTCCCTTTGTCATCCT-3’	
	Reverse	5′- GTGCAGGGTCCGAGGT-3’	
PARP1	Forward	5′-GACGTGGAGAGCATGAAGAAGG-3’	(Designated)
	Reverse	5′-GGTGTAGAAGCGATTGGAGAGA-3’	
HIF1α	Forward	5′-CAAAGACAATAGCTTTGCAGAATG-3’	[24]
	Reverse	5′-ACGGTCACCTGGTTGCTG-3’	
NFATc2	Forward	5′-GGCAGCAGATTTGGGAGATGG-3’	(Designated)
	Reverse	5′-GTGCTGTGGGTAATATGGCTGG-3’	
a-SMA	Forward	5′-CCTGGCTTCGCTGTCTACCT-3’	[25]
	Reverse	5′-TTGCGGTGGACGATGGA-3’	
RNU6	Forward	5′-CTCGCTTCGGCAGCACA-3’	[26]
	Reverse	5′-AACGCTTCACGAATTTGCGT-3’	
18 s	Forward	5′-AGTCCCTGCCCTTTGTACACA-3’	[27]
	Reverse	5′-GATCCGAGGGCCTCACTAAAC-3’	

Fold change gene expression for miRNA-204 and target genes was calculated according to the formula: Fold change = 2^–ΔΔCT^ in which ΔΔCT is the difference between the DCT of each group and the DCT of the CTL group, with DCT equal to CT gene minus CT internal control. Therefore, ΔΔCT = [(CT gene - CT RNu6) _treatment_ – [CT gene - CT RNu6)] _CTL._

### Western blot analysis

Total protein was extracted from homogenized lung tissues with a protease inhibitor cocktail (Sigma-Aldrich, USA) in ice-cold RIPA buffer (Cytomatingene) and then centrifuged at 15000 rpm for 10 min at 4 °C, and the supernatant was collected. The protein concentration in the supernatant was assessed by the Lowry method [28]. Then, the samples were separated by polyacrylamide gel electrophoresis (Bio-Rad, USA) via 4–20% gradient polyacrylamide gels containing 0.1% sodium dodecyl sulfate for ~ 2 h at 95 V and then transferred to PVDF membranes (Carl Roth, Germany) for 80 min at 80 V. The membranes were blocked overnight at 4 °C in TBS containing Tween and 5% fat-free milk (Sigma-Aldrich, St. Louis, USA). The protein expression of HIF1α, PARP1, NFATc2, and GAPDH (served as a loading control) were determined. Then membranes were washed extensively with PBS-Tween and incubated with secondary antibodies for 1 h at room temperature. After washing, membranes were developed using DAB (3, 3′-diaminobenzidine) substrate, and their images were captured and analyzed using Image J software. The blots for animal groups are presented in supplementary figure 1.

### Statistical analysis

Data were expressed as mean ± SEM. The normal distribution of data was verified using the Kolmogorov-Smirnov test and then analyzed using one way ANOVA test to assess the difference among the groups. In the case of significance the Tukey’s post-hoc test was used to explore the significantly different groups. Nonparametric data were analyzed by the Kruskal-Wallis test followed by Mann-Whitney U test as post-hoc. *P* values less than 0.05 were considered statistically significant.

## Results

### Determining optimum doses of PA and QS

To determine the optimum doses of PA and QS in treatment of PAH, after induction of the disease, a pilot dose-response study was performed using different doses of these plant derivatives (Fig. 1). Based on the ameliorative effects on arteriole wall thickness, the lowest dose with maximum effect was 50 mg/kg for PA and 30 mg/kg for QS. For the rest of the study only these doses were used.

### Effect of PA and QS on RVSP and ventricular hypertrophy

Table 2 contains the results of RVSP and RV hypertrophy index measurements in the studied groups. MCT increased both indices significantly (*p* < 0.001), and vehicle treatment had no therapeutic effect. PA (50 mg/kg) and QS (30 mg/kg) treatment resulted in significant reduction of both indices towards normal level (*p* < 0.001).

**Table 2:** Right ventricular systolic pressure (RVSP) and RV/ LV + Septum weight ratio values in monocrotaline (MCT)-induced pulmonary hypertension, and the effect of treatment with perillyle alcohol (PA) and quercetin (QS)

Groups	RVSP	RV/ LV + septum ratio
CTL	29.5 ± 1.5	0.31 ± 0.03
MCT	90.7 ± 4.6^***^	0.61 ± 0.04^***^
MCT + Veh	91.8 ± 5.4^***^	0.56 ± 0.03^***^
MCT + PA	34.8 ± 4.1^###^	0.34 ± 0.04^###^
MCT+ QS	50.8 ± 16.6 ^###^	0.36 ± 0.04^###^

*** *p* < 0.001 compared to CTL, and ### p < 0.001 compared to MCT + Veh group. *n* = 6 in each group

### Histopathology of the lung

**Fig. 2:**
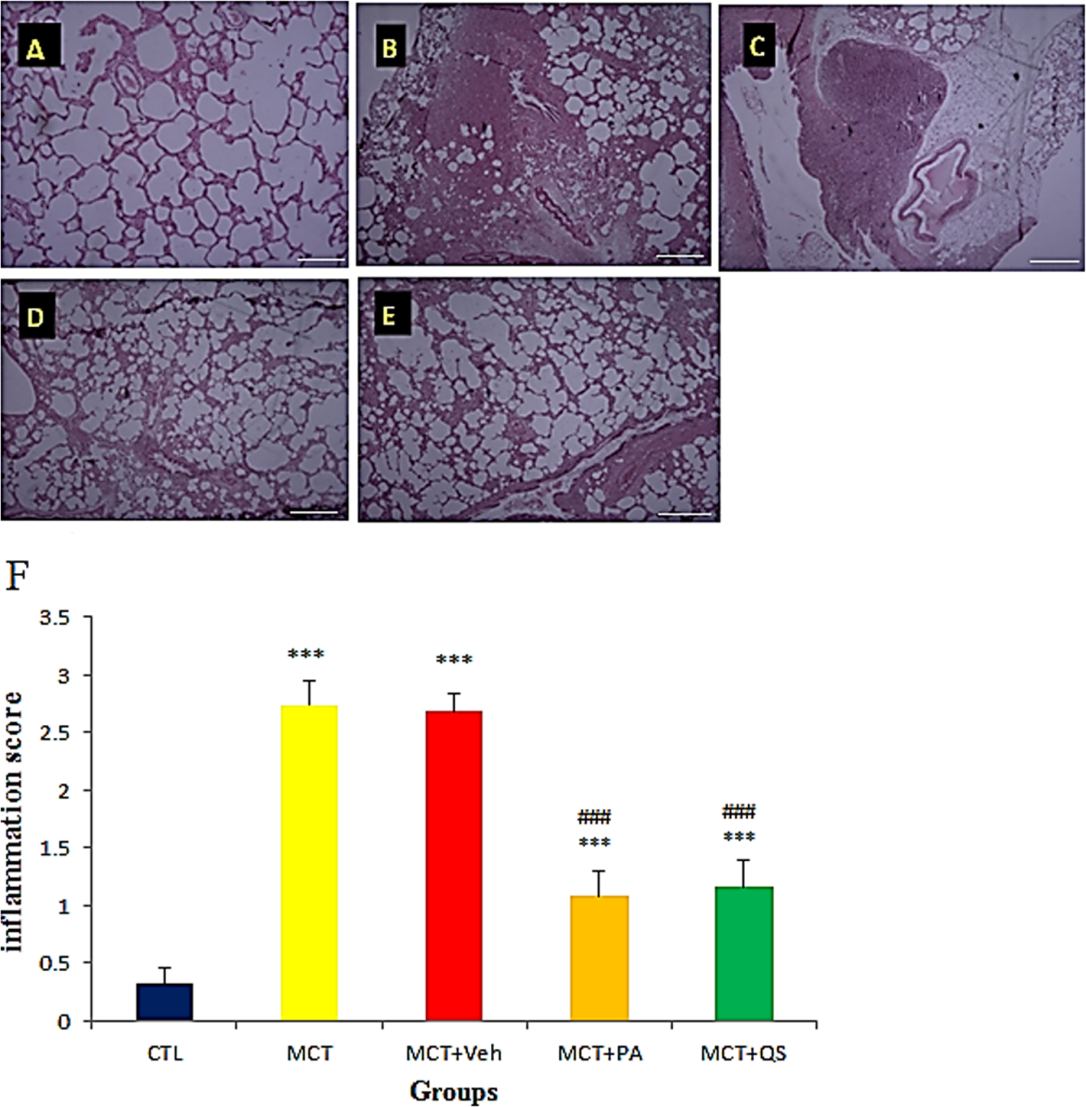
Representative section of the lung of a normal rat (**a**), MCT-induced PAH (**b**), MCT + vehicle treatment (**c**), MCT + PA treatment (**d**) and MCT + QS treatment (**e**). **f:** relative quantitative analyses (mean ± SEM) of groups A-E. H&E Staining; magnification X 100. (*n* = 6 in each group). PA, Perillyle alcohol and QS, Quercetin. Scale bars are 20 μm. *** p < 0.001 vs control, ### p < 0.001 vs MCT + Veh

In control rats, the lung histology was normal. Severe inflammation was observed in MCT and MCT + vehicle (Veh) lung tissues (Fig. 2). Treatment with PA and QS decreased inflammation significantly (*p* < 0.001).

**Fig. 3:**
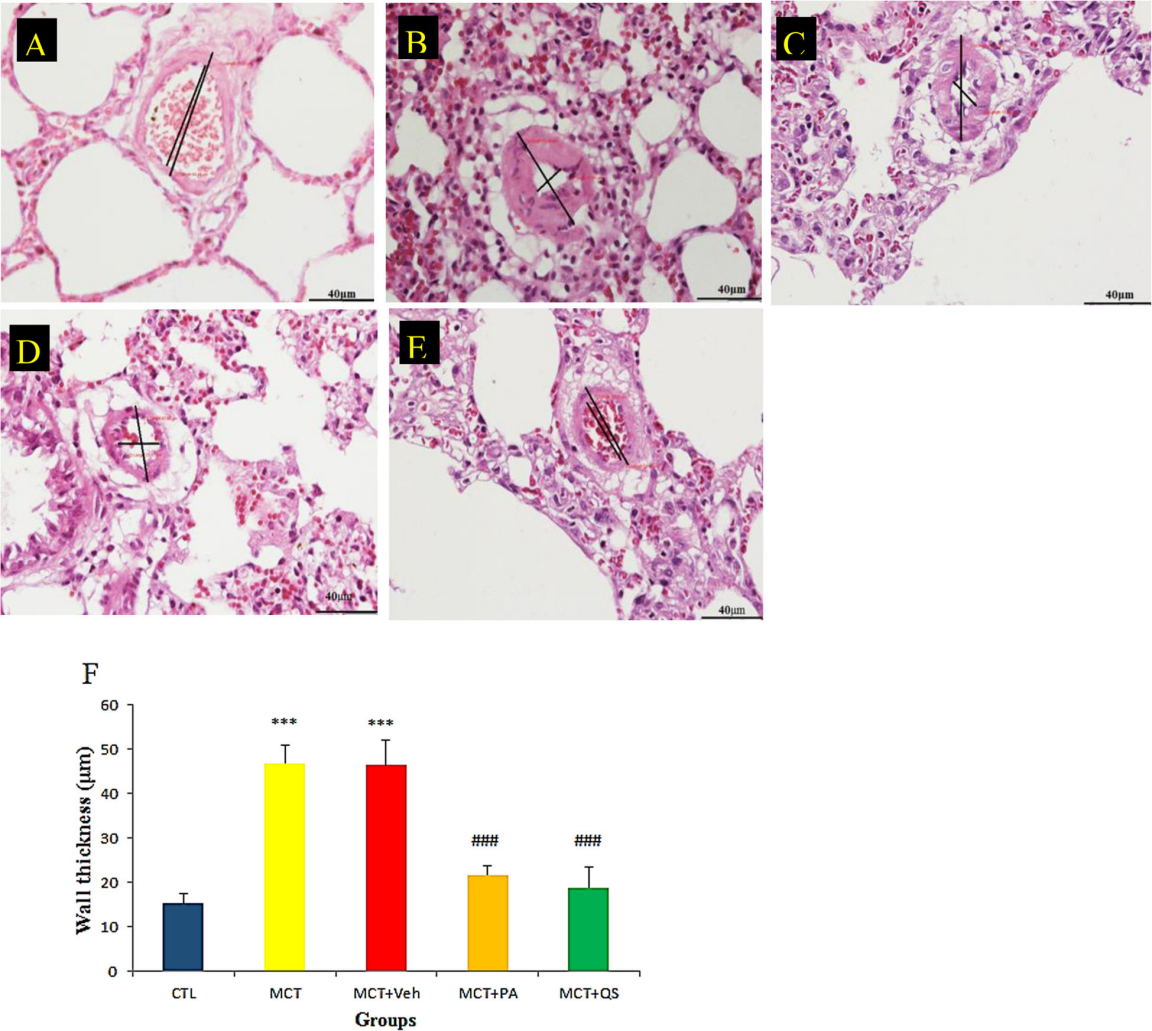
Representative section of the lung of a normal rat (**a**), MCT induced PAH (**b**), MCT + vehicle treatment (**c**), MCT + PA treatment (**d**), and MCT + QS treatment (**e**), and (**f**) relative quantitative analyses (mean ± SEM) in groups A-E. Normal wall thickness is observed in A, and severe thickening is obvious in B and C. The lung of rats treated with PA and QS show mild thickening of arteriole wall. Here arteriole wall thickness in the CTL group is 14.3 μm, 44 μm in MCT, 43 μm in vehicle, and 18.2 μm and 15.3 μm in PA and QS, respectively. H&E staining; Magnification × 400. *n* = 6 in each group. Scale bars are 40 μm. *** *p* < 0.001 vs control, ### p < 0.001 vs MCT + Veh. CTL, control; MCT, monocrotaline; VE, vehicle; PA, perillyle alcohol; QS, quercetin

Figure 3 shows the representative sections of the lungs of the studied groups regarding arteriole wall thickening. As for inflammation, the arteriole wall thickness was normal in the control group, severely thickened in MCT and MCT + Veh, and reduced significantly towards normal in MCT + PA and MCT + QS groups.

### Effect of PAH on α-SMA and response to PA and QS treatment

**Fig. 4:**
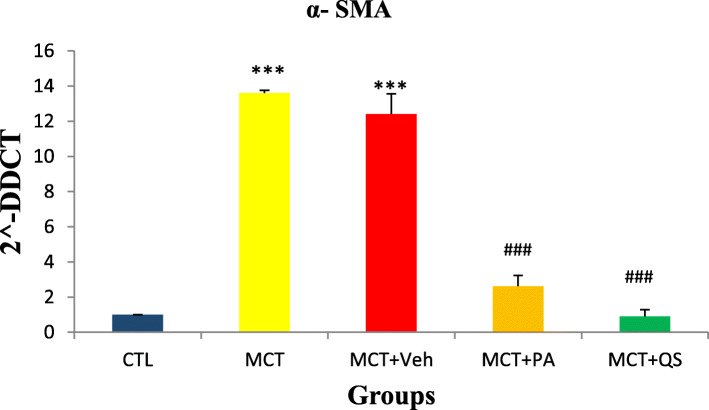
Relative expression (Mean ± SEM) of α-SMA mRNA in the lungs of PAH rat models, and the effect of treatment with PA and QS. *n* = 6 in each group. *** and ### = P < 0.001, significantly different with both control and MCT + VE. CTL, control; MCT, monocrotaline; VE, vehicle; PA, perillyle alcohol; QS, quercetin; α-SMA, α-smooth muscle actin

We considered mRNA expression of α-SMA in the lungs of PAH rat models as an index of smooth muscle cell proliferation. Figure 4 shows significant increase in the expression of α-SMA mRNA compared to control group (*P* < 0.001). PA and QS treatment recovered the increments towards normal level (p < 0.001). There was no significant difference between the PA and QS groups.

### Effect of PAH on inflammatory cytokines and response to PA and QS treatment

**Fig. 5:**
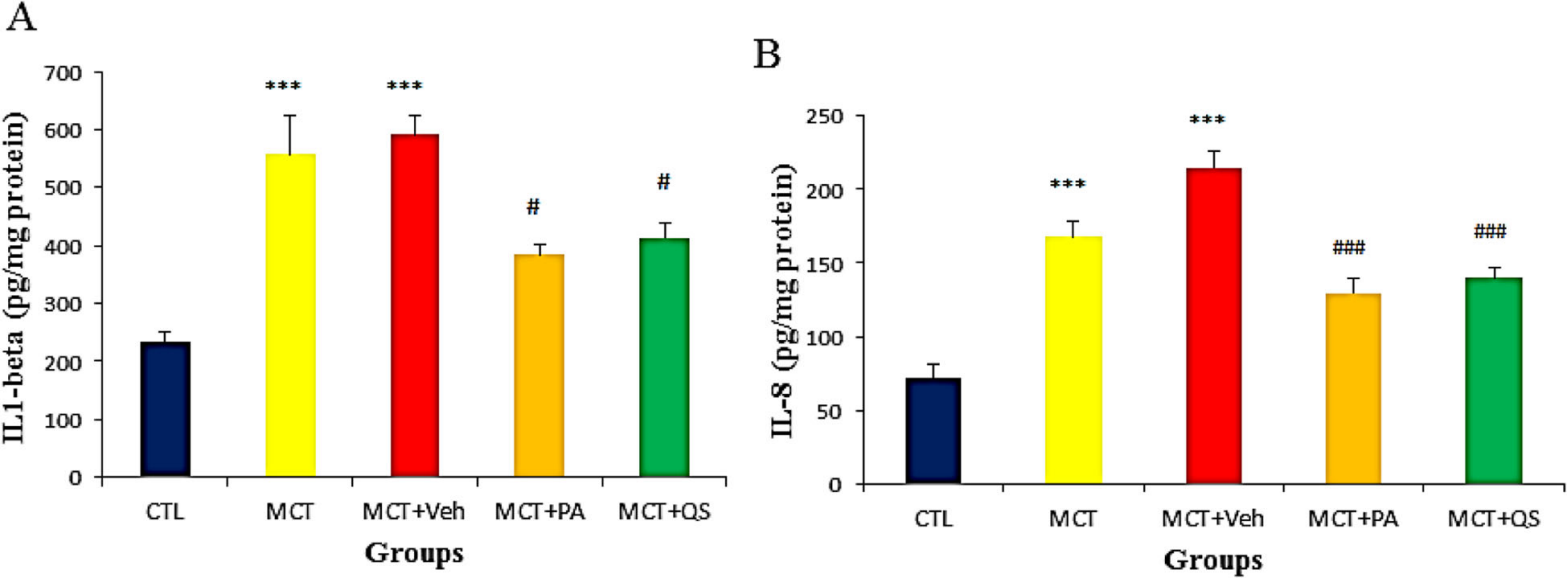
The expression of inflammatory cytokines IL-1β (**a**) and IL-8 (**b**) in monocrotaline-induced PAH lungs and the effect of treatment with PA and QS. *n* = 6 in each group. *** *p* < 0.001 vs Control, # p < 0.05, ### p < 0.001 vs MCT + Veh. CTL, control; MCT, monocrotaline; Veh, vehicle; PA, perillyle alcohol; QS, quercetin

The level of IL-1β and IL-8 increased in MCT groups and treatment with PA and QS decreased them significantly (*p* < 0.05 and *p* < 0.001, respectively). There was no difference between the effect of PA and QS (Fig. 5).

### Effect of PAH on miR-204 and PARP1 expression and response to PA and QS treatment

**Fig. 6:**
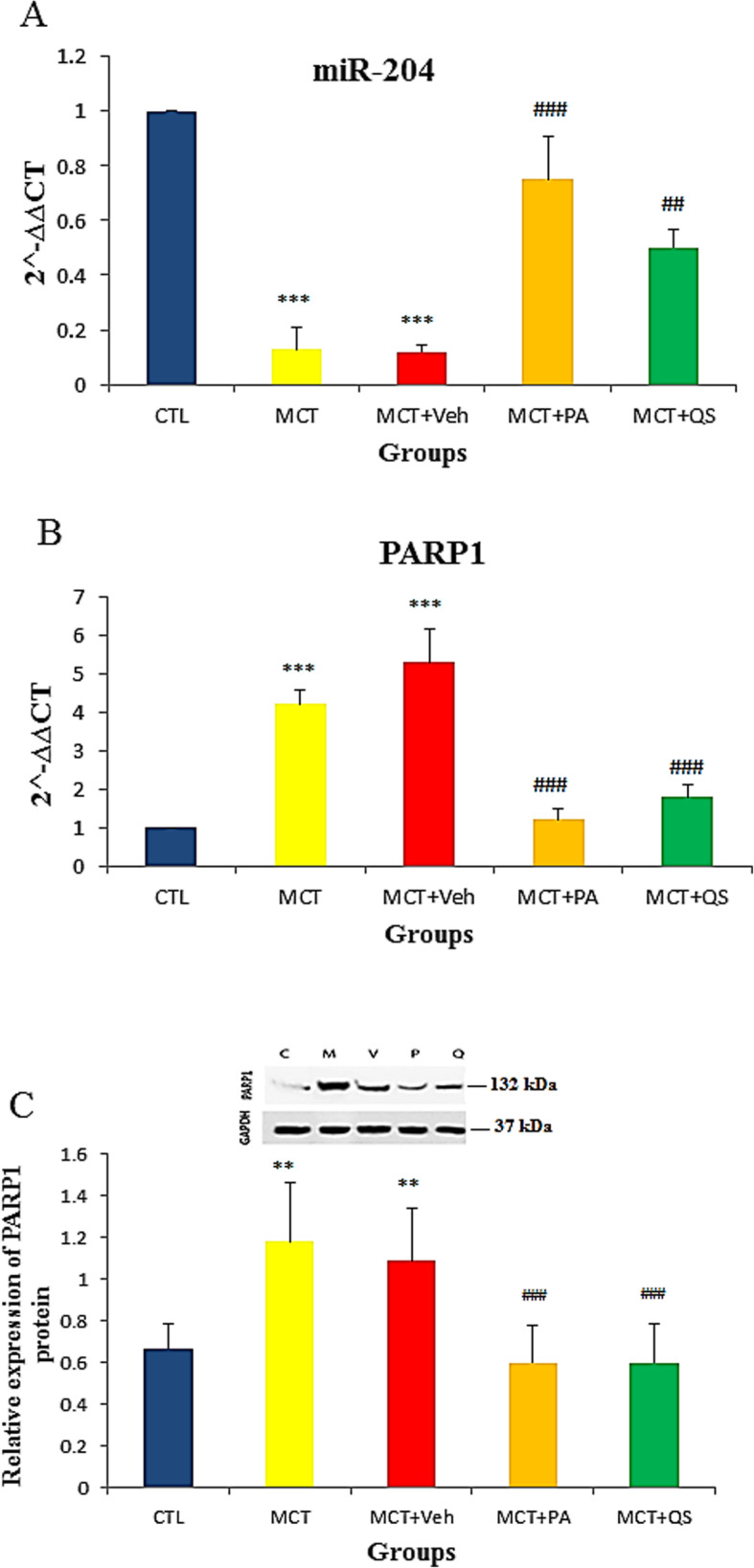
Relative expression (Mean ± SEM) of miR-204 (**a**), PARP1 mRNA (**b**) and PARP1 protein (**c**) in the lungs of studied groups. *n* = 6 in each group. The internal controls for PARP1 mRNA and miR-204 mRNA were 18 s rRNA and RNA U6 (RNU6), respectively. ** p < 0.01, *** p < 0.001 vs Control, ## p < 0.01, ### p < 0.001 vs MCT + Veh. CTL, control; MCT, monocrotaline; Veh, vehicle; PA, perillyle alcohol; QS, quercetin; PARP1, Poly ADP-ribose polymerase-1

The level of miR-204 expression in the lung tissues of rats treated with MCT + vehicle significantly decreased compared to normotensive (CTL) rat lungs (*p* < 0.001) (Fig. 6). PA and QS treatment significantly recovered the MCT-induced reduction in miR-204. Both mRNA (*p* < 0.001) and protein (*p* < 0.01) expressions of PARP1 increased in PAH rat models. These increments were also significantly recovered by PA and QS treatment (Fig. 6b and c). There was no significant difference between the effects of PA and QS.

### Effect of PAH on HIF1α and NFATc2 expression and response to PA and QS treatment

**Fig. 7:**
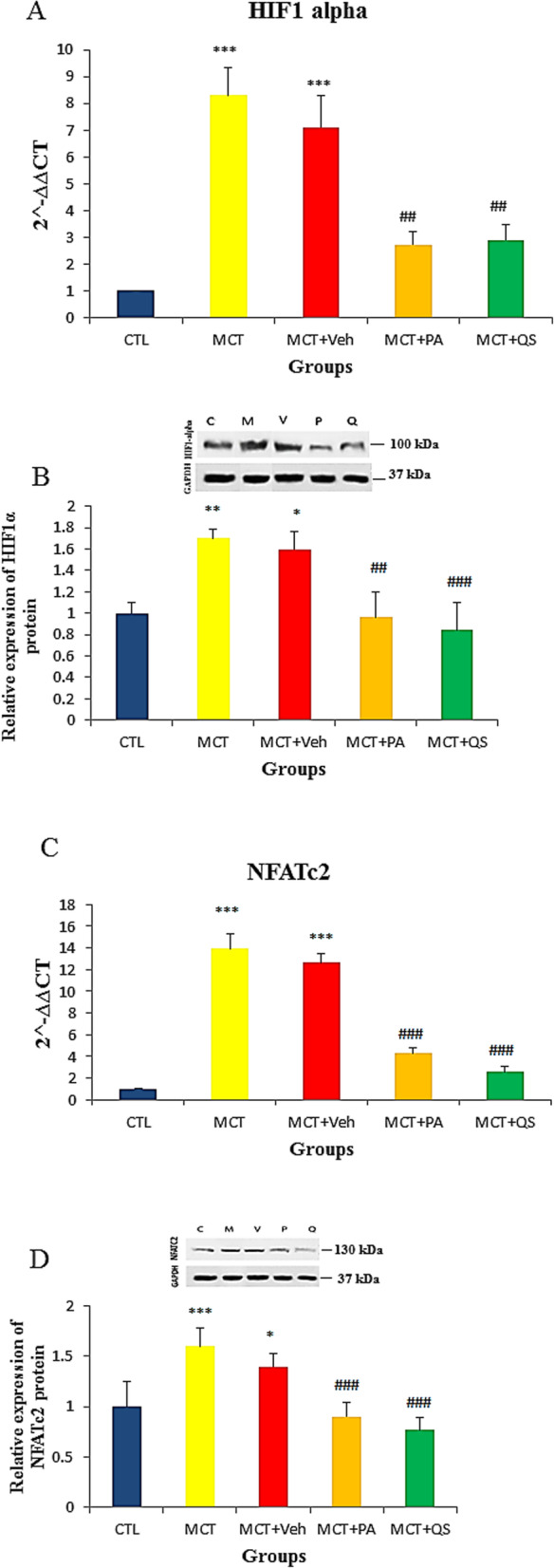
Relative expression (Mean ± SEM) of HIF1α and NFATc2 mRNA (**a**, **c**) and protein (**b**, **d**) in the lungs of PAH rat models, and the effect of treatment with perillyle alcohol and quercetin. *n* = 6 in each group. Internal control for HIF1α and NFATc2 mRNA was 18 s rRNA. *and # mean significantly different with control and MCT + Veh respectively. * *p* < 0.05, ** and ## *p* < 0.01, *** and ### *p* < 0.001. CTL, control; MCT, monocrotaline; Veh, vehicle; PA, perillyle alcohol; QS, quercetin; HIF1α, hypoxia-inducible factor 1-alpha; NFATc2, nuclear factor of activated T-cells, cytoplasmic 2

Figure 7 shows significant increase in the expression of mRNA and protein of HIF1α and NFATc2 in the lungs of PAH rat models. PA and QS treatment significantly recovered the increments in the expression of mRNA and protein of HIF1α and NFATc2 towards normal level. There was no significant difference between the effects of PA and QS.

## Discussion

In this study, we investigated the effect of PA and QS on miR-204 expression and its downstream protein targets in rat lungs affected by MCT-induced PAH. The main findings of the study were: 1- miR-204 expression decreased in PAH rat models and PA and QS recovered this reduction in miR-204 expression. 2- The expression of mRNA and protein of PARP1, HIF1α, and NFATc2 increased significantly in the lung of PAH rat models and these were reduced to almost control level by treatment with PA and QS. The underlying mechanisms may be increase in the expression of PARP1 followed by reduction of miR-204.

MiR-204 is an important miRNA in many diseases including different types of cancer and PAH [11, 29, 30]. Recent studies have shown that some miRNAs, such as miR-204, can be risk factors for patient longevity. For example, in patients with myeloid leukemia (AML), it was identified that when the expression of miR-204 gene is low the patient’s survival is shorter [30]. The recovery in miR-204 by PA and QS accompanied by reduction in arteriole wall thickness and inflammation in the lung of PAH rat models (Figs. 2 and 3) showed that the ameliorative effect of these plant derivatives on PAH may be through their anti-inflammatory and anti-proliferative properties. PA and QS reduced inflammation and proliferation in lungs with PAH equipotently. Previous studies reported that miR-204 is involved in processes such as inflammation, apoptosis, proliferation, invasion, and migration [8, 10] . In a study on HK-2 renal tubular epithelial cells, it was indicated that miR-204 can suppress inflammation by binding to the IL-6 receptors [31]. In another study it was found that the protein Bcl2, which has anti-apoptotic effects, is an miR-204 target [32].

Our results showed that the expression of PARP1 increased in rat lungs with PAH and decreased after treatment with PA and QS. In agreement with this finding, the elevation of PARP1 in human PAH pulmonary arteries and cultured PAH-PASMC have been associated with reduction in the expression of miR-204 [9]. PARP-1 is the most abundant sensor and effector that responds to a wide variety of stress signals, including inflammatory, oxidative, and metabolic stress in pathological conditions such as cancer and PAH [33]. PARP-1 is a critical enzyme functioning at the center of cellular stress responses implicated in DNA repair, allowing proliferation, despite the presence of DNA-damaging insults in these conditions. PARP performs most of its functions through NAD as an ADP ribose [9, 33]. In PAH, PARP repairs damaged DNA and also activates the NFAT and HIF1 pathways leading to exacerbation of inflammation [9]. Therefore, inflammation found in the present study in the lungs of PAH rat models can be an inducing factor for the increase in the expression of PARP1.

Down-regulation of miR-204 activates proliferative and anti-apoptotic pathways related to NFATc2 and HIF1α in lungs in rat models of PAH. According to our results, it seems that treatment of MCT-induced PAH rat models with PA and QS has reduced the proliferation of PASMCs by reducing the expression of PARP1, subsequently increasing the expression of miR-204, and reducing NFATc2 and HIF1α in the lungs. The high level of α-SMA expression which is a hallmark of extracellular matrix (ECM) remodeling in PAH rat models [33] verifies the proliferative effect of PAH in lung arteriole smooth muscles. Alpha-SMA is generally considered as a factor to measure pulmonary arterial smooth muscle cell (PASMC) presence and activity [34]. Again treatment with PA and QS significantly suppressed this effect of the disease.

It has been reported that inhibition of NFATc2 by cyclosporine (indirect inhibitor of NFAT) can reverse MCT-induced PAH in rats [34], and that resistance to apoptosis in PASMCs is characterized by mitochondrial hyperpolarization and activation of the NFAT [35]. NFATc2 down regulates K+ channels, especially Kv1.5, which inhibits apoptosis [36]. On the other hand, Kv1.5 down regulation causes an influx of Ca++, which in turn promotes proliferation and vasoconstriction [12].

Also several studies have shown that resistance to apoptosis in PAH occurs due to abnormal mitochondrial function under the influence of HIF1α [9, 37]. HIF-1α is activated by ROS and has been shown, to induce high levels of vascular endothelial growth factor (VEGF) expression, thereby promoting proliferation [12]. Also HIF-1α activation, similar to NFAT, down regulates Kv1.5 channels, promoting proliferation and resistance to apoptosis [12, 37]. Another study in rats showed that inhibition of HIF1α in vitro and in vivo reduces cell proliferation and vascular remodeling [38]. Thus, probably the reduction in the expression of NFAT and HIF1α, which are downstream signaling pathways of miR-204-PARP1, may have played a role in the ameliorative effect of PA and QS on PAH found in the present study. Almost in all results of the present study PA and QS equipotently showed ameliorating effect on PAH. In the present study we did not assess the impact of the combination of these two phytochemicals. We propose an isobolographic study to determine the type of interaction between these two plant derivatives and/or find the best dose ratio of their combination in the treatment of PAH.

## Conclusion

PA and QS improved PAH possibly through affecting the expression of PARP1 and miR-204 and their downstream targets, HIF1a and NFATc2. According to the results, we recommend that PA and QS may be therapeutic goals in the treatment of PAH as these plant derivatives are naturally present in our daily diet and are thus bio-compatible with the human body.

## Supplementary information

**Additional file 1 : Supplementary Figure 1.**The western blots for animal groups. *n* = 5 C = Control, V = Vehicle, P = Perillyle alcohol, Q = Quercetine, M = Monocrotaline. The data related to B is not presented in the Paper.

## Data Availability

The datasets used and/or analyzed for this study will be available from the corresponding author by request.

## Abbreviations

PAH: Pulmonary artery hypertension
MCT: Monocrotaline
PAP: Pulmonary arterial pressure
PASMCs: Pulmonary arterial smooth muscle cells
CTL: Control
Veh: Vehicle
PA: Perillyle alcohol
QS: Quercetin
PARP1: Poly ADP-ribose polymerase-1
HIF1α: Hypoxia-inducible factor 1-alpha
NFATc2: Nuclear factor of activated T-cells cytoplasmic 2
