# Supplementary material for: Perillyle alcohol and Quercetin ameliorate monocrotaline-induced pulmonary artery hypertension in rats through PARP1-mediated miR-204 down-regulation and its downstream pathway
Source: BMC Complement Med Ther. 2020 Jul 13;20:218. doi: 10.1186/s12906-020-03015-1 (PMC7359282; doi:10.1186/s12906-020-03015-1)

**Supplementary fig 1**.The western blots for animal groups. n=5 C= Control, V= Vehicle, P= Perillyle alcohol, Q= Quercetine, M= Monocrotaline. The data related to B is not presented in the Paper.


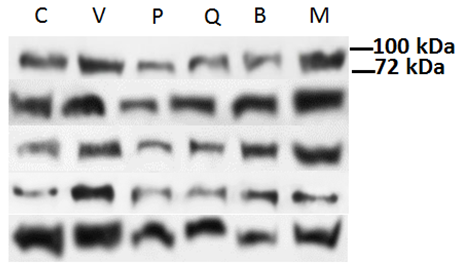

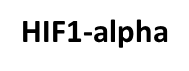

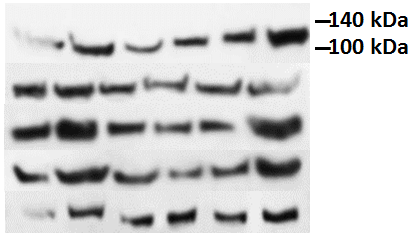

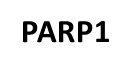

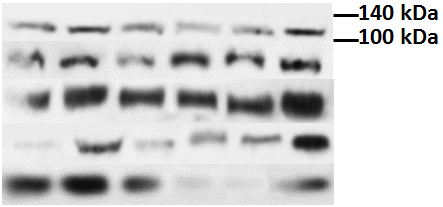

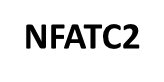

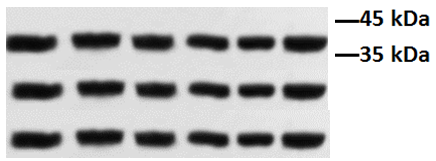

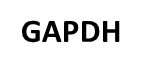

Supplement: Supplementary file 1 — Additional file 1 : Supplementary Figure 1.The western blots for animal groups. n = 5 C = Control, V = Vehicle, P = Perillyle alcohol, Q = Quercetine, M = Monocrotaline. The data related to B is not presented in the Paper. [file 12906_2020_3015_MOESM1_ESM.docx]
